# Mechanical properties and crack evolution characteristics of fractured rock with hidden fissures

**DOI:** 10.1038/s41598-023-38285-5

**Published:** 2023-07-19

**Authors:** Yuxin Ban, Lihong Chen, Qiang Xie, Jun Duan, Chunbo He, Xiaoqiang Xue, Xiang Fu

**Affiliations:** 1 School of Civil Engineering and Architecture, Chongqing University of Science and Technology, Chongqing, 401331 China; 2 School of Civil Engineering, Chongqing University, Chongqing, 400044 China; 3 National Joint Engineering Research Center for Prevention and Control of Environmental Geological Hazards in the TGR Area, Chongqing, 400044 China; 4 College of River and Ocean Engineering, Chongqing Jiaotong University, Chongqing, 400074 China

**Keywords:** Civil engineering, Petrology

## Abstract

Natural defects, such as joints, structural surfaces and voids, significantly affect the mechanical properties and fracture modes of rock mass. Hidden fissures are widely distributed in magmatic rock, while their influences on the mechanical properties and the cracking mechanism are still unclear. Laboratory tests were conducted on prefabricating hidden-fissured rock-like specimens, as well as intact specimens and close-fissured specimens as a comparison. The real-time digital image correlation technology and acoustic emission monitoring technology were synchronously adopted to capture both the external and internal cracking process. The results show that the hidden fissures can weaken the uniaxial compression strength, while the deterioration effect of hidden fissures is weaker than closed fissures due to the internal cohesion among fissure internal particles. What’s more, the initiation behavior of the *α* = 90° hidden-fissured specimen is different from that of *β* = 90° closed-fissured specimen. Finally, the cracking mechanism of hidden-fissured specimens was revealed by analyzing the RA–AF relationship. The failure of the close-fissured specimens is mainly the tensile-shear mixed fracture mode, while the failure of the hidden-fissured specimens is mainly the tensile fracture mode and supplemented by the shear. The experimental results contribute to the understanding of cracking properties in hidden-fissured rock.

## Introduction

**Figure 1:**
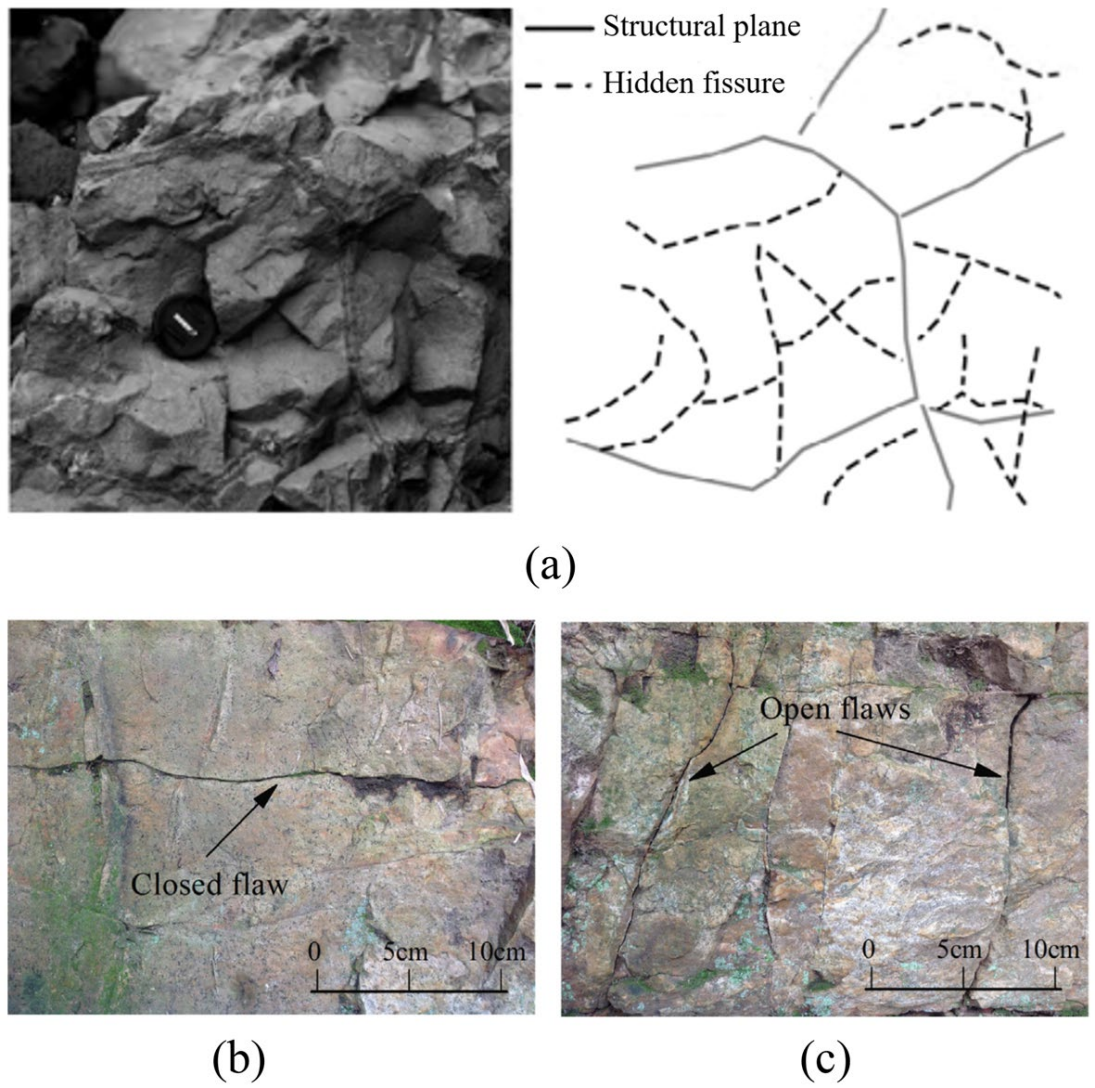
Natural flaws in rock mass: (**a**) hidden fissures in columnar basalt^8^, (**b**) closed fissure and (**c**) open flaw^3^.

Rock mass in nature contains various kinds of discontinued planes, such as faults, joints and fissures, under the effect of long-term geological processes. These geological discontinuities make the mechanical properties of rock mass quite different from intact rock^1,2^. Especially for magmatic rock, the cooling and shrinking effects after magmatic exhalation contribute to plenty of hidden fissures in rock mass^3^, as shown in Fig. 1a. In Southwest China, many hydropower projects, including Xiluodu, Baihetan and Wudongde Hydropower Stations, are built on the magmatic rock^4–7^. The mechanical properties of magmatic rock with hidden fissures significantly influence the surrounding rock stability of rock engineering^8^. Therefore, understanding the influence of hidden fissures on the mechanical behavior of rock mass is the key to evaluating the stability of rock engineering, and the study can also provide support for geotechnical engineering design and construction.

In uniaxial compression, failure of intact rock specimens is mainly caused by tensile cracks or shear cracks. The cracks gradually develop in the direction parallel to the maximum principal stress, resulting in the fracture mode of the specimen evolving from shear failure to tensile failure as axial stress increase^9^. Obviously, the existence of defects in the specimen will significantly reduce the mechanical parameters of the specimen, which in turn affects the crack type and fracture mode. In the past several decades, the mechanical properties and cracking behaviors of fractured rock mass have been widely studied. In general, these studies focused on rock mass containing closed fissures or open fissures. As shown in Fig. 1b,c, the difference between closed and open fissures is whether there is contact and friction on fracture surfaces^10^.

For example, Wong et al.^11^ used marble and PMMA rock-like materials to study the crack propagation of different defect depths and dip angles, and found that the failure mechanism of PMMA specimens was similar to that of marble specimens. Ghazvinian et al.^12^ used low-brittle rock-like materials to make Brazilian disc specimens with prefabricated fissures and studied the mixed fracture mode of cracks by Brazilian splitting test. Zhuang et al.^13^ divided the propagation forms of main cracks in rock or rock-like specimens with single prefabricated cracks into three types: secondary cracks, airfoil cracks and anti-foil cracks. Jin et al.^14^ conducted laboratory tests and numerical simulations on artificial rock models with closed fissures to study the influence of a single fissure on strength, failure process and energy consumption. According to the failure mechanism of cracks, Xu^15^ conducted uniaxial compression tests on gypsum specimens containing a single closed defect with different inclination angles, and analyzed in detail the effects of crack orientation on strength, cracking mechanism, fracture mode and acoustic emission (AE) behavior. Meng et al.^16^ studied the comprehensive effects of different bedding plane angles and notch angles on the mixed-mode fracture behavior of rock-like specimens by using AE technology. In addition, with the number of fissures increases, the crack initiation position, coalescence trajectory and fracture mode become more complex. For example, Wong et al.^17^ conducted experimental research on rock-like specimens containing three parallel cracks, and they found that the arrangement of defects and the friction coefficient of the defect surface affect the healing mechanism of cracks, and the peak strength is related to the number of cracks. Sagong and Bobet^18^ conducted uniaxial compression tests on gypsum specimens containing three prefabricated defects and 16 prefabricated defects, respectively. The results showed that the cracking mode of multi-defect specimens was similar to that of double-defect specimens. Park and Bobet^19^ tested the closed fissure gypsum specimens with different angles, spacing and continuity. It was observed that the crack types of open defects and closed defects were the same, and the coalescence types were similar. Zhou et al.^20^ conducted experiments on rock-like specimens with four cracks to study the effects of multi-crack layout on mechanical properties, crack initiation modes and crack coalescence types, from which five types of cracks and ten types of crack coalescence modes were found. Cao et al.^21^ loaded rock-like specimens with two pre-existing defects, observed different geometric shapes of cracks, and determined seven types of coalescence. From the microscopic point of view, Luo et al.^22^ studied the influence of three different fillers on the fracture morphology and failure behavior of fissure-filled rock-like specimens under compression-shear load. Zhao et al.^23^ used the volume loss method to prepare gypsum rock-like specimens with various internal open defects, and combined with acoustic emission technology to study the effects of different defects on the mechanical properties and failure characteristics of hard brittle rock specimens.

These studies reveal the influences of number, geometry and filling material on the mechanical and failure mechanism of rock specimens containing open or closed fissures, laying an important foundation for developing fractured rock mass mechanics. However, the hidden fissure is significantly different from a closed fissure or open fissure, as the fracture surface offers friction and cohesive force as the mineral particles still coalesce into a whole section. Although the hidden fissures are difficult to observe without external force interference, they will be exposed when unloading occurs, and the influences should not be ignored. The effects of hidden fissures on the mechanical properties and cracking behaviors of rock mass are rarely studied.

To this end, taking the intact and close-fissured specimens as a comparison, the evaluation of mechanical parameters and cracking properties of the hidden-fissured specimen with fissure angles are tested. The experimental process is introduced in "Experimental methods", and the mechanical properties and cracking behaviors of hidden-fissured and close-fissured specimens are given in "Hidden-fissured specimens" and "Close-fissured specimens", respectively. In “Discussion”, the strength reduction induced by the hidden fissures is analyzed. Furthermore, the failure mechanism is revealed by the acoustical method.

## Experimental methods

### Specimens fabrication

**Figure 2:**
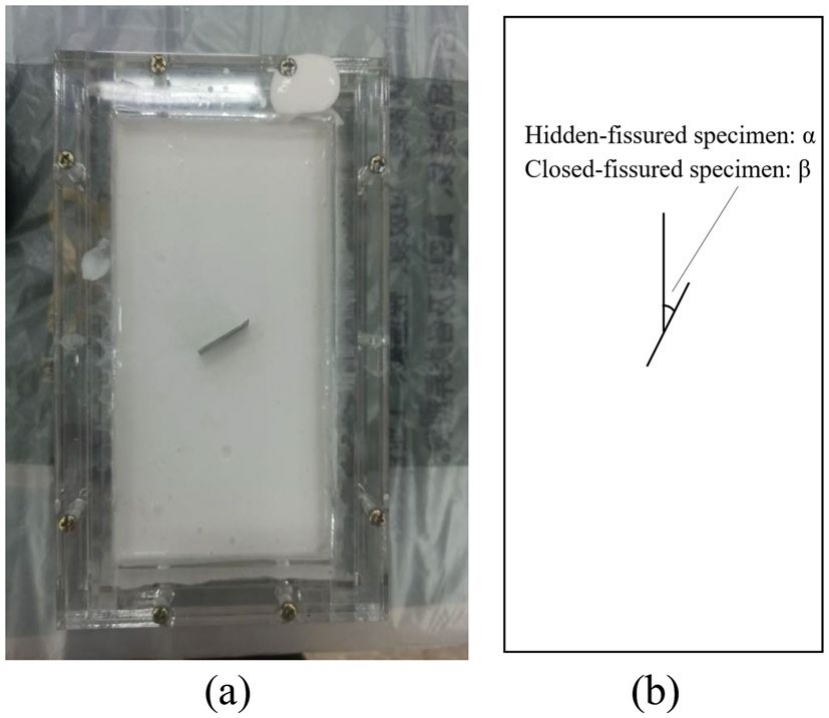
(**a**) The fabrication process of the fissured specimens and (**b**) schematic diagram of the fissure angle.

High-strength gypsum is adopted to fabricate rock-like magmatic specimens^10,24^. Combined similarity law calculation with pre-experiments, the mass ratio of similar materials and water is 3:1. The mixture is poured into transparent boxes to form 50 mm × 100 mm rectangular specimens after solidification, as shown in Fig. 2a. Meanwhile, the fissures were prefabricated by thin slice extraction method^19,25^. A rectangular strip steel sheet (1 mm × 20 mm × 50 mm) with enough strength and stiffness is put in the box middle immediately after the mixture is poured.

It should be noted that the key to obtaining hidden-fissured specimens is to control the pull-out time of thin steel sheets. If the thin steel sheet is pulled out too early, the fissure may disappear due to material fluidity; on the contrary, a closed fissure will be formed as the material is solidified if the thin steel sheet is pulled out too late. The pulling-out time of the hidden-fissured specimen should be shorter than that of the close-fissured specimen. After several repeated attempts, it is found that a hidden-fissured specimen can be obtained by pulling it out after 10 min. Lubricant is smeared on the steel sheet for close-fissured specimen, and setting the pulling-out time to 18 min or even longer. The close-fissured specimen is obtained after the material is totally solidified. The angle between the hidden fissure (closed fissure) and vertical direction is *α* (*β*) = 0°, 30°, 60° and 90° (Fig. 2b).

### Experiment system

**Figure 3:**
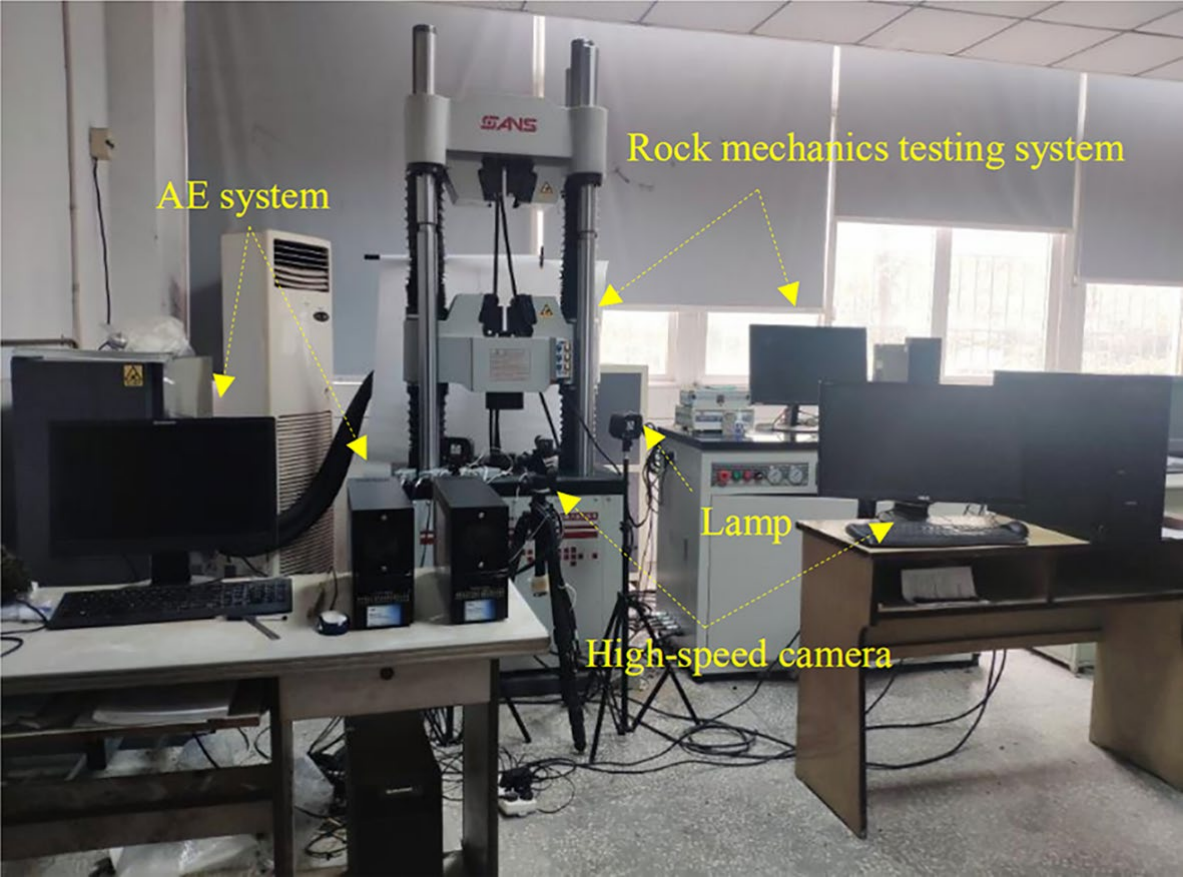
The experiment system is composed of loading module, image acquisition module and AE monitoring module.

The experiment system comprises a loading module, image acquisition module and acoustic emission (AE) monitoring module, as shown in Fig. 3. The specimens are loaded on SHT465 Rock Mass Mechanics Test Machine. The loading mode is controlled by force, and the loading rate is set to 50 N/s. The image acquisition system uses a Basler acA2440-75um industrial camera with a resolution of 2048 × 2048 pixels and a shooting rate of 10 frames/s to monitor the deformation and cracking process of the specimen. The Vic-Snap8 commercial software program is used to calculate the surface displacement field and strain field. In addition, two LED lights are used to illuminate the surface of the specimen. An artificial speckle field is made on the specimen front surface for digital image correlation (DIC) analysis. Acoustic emission monitoring is conducted via an 8-channel PCI-II instrument from American Physical Acoustics Company. It can continuously collect and automatically store AE parameters and waveform data in real-time. The threshold value is set to 40 dB and the sampling frequency is 1MSPS. Nano-30 AE sensors are coated with a coupling agent and placed on the specimen’s surfaces. The preamplifier is set to 40 dB.

Vaseline is used between the specimen ends and loading plates to reduce the end effect. The loading module, image acquisition module and AE monitoring module start synchronously to acquire and record data.

## Test results

### Hidden-fissured specimens

#### Stress–strain relationship

**Figure 4:**
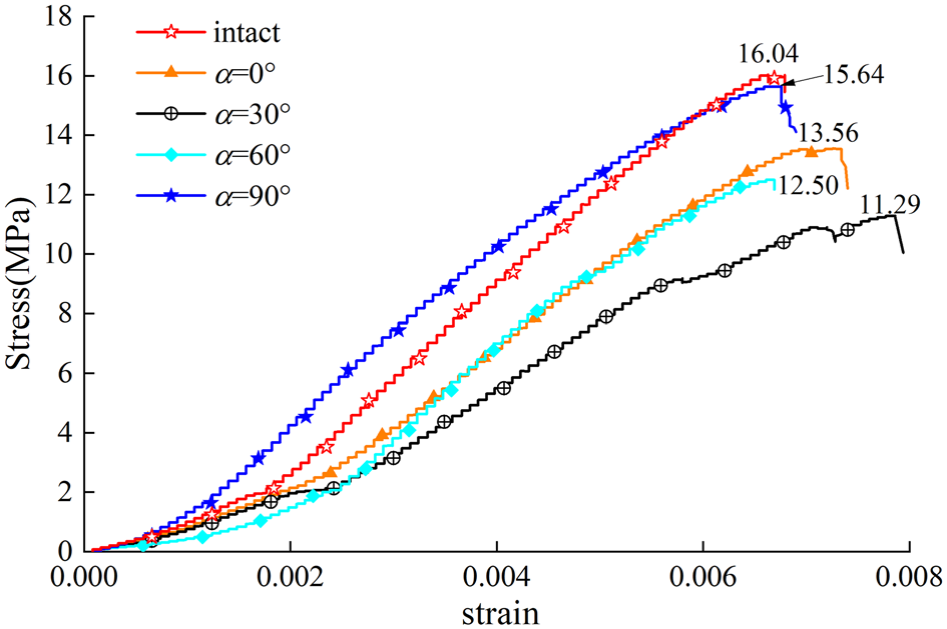
Stress–strain curves of specimens with different hidden fissure orientations.

Figure 4 shows the stress–strain curves of hidden-fissured specimens with different fissure inclination angles and intact specimens. All specimens undergo similar stress–strain curves evolution processes, i.e., compression, elastic and damage stages. The curves suddenly drop after rising to peak due to rapid energy release from cracks. Among all specimens, the slope of the intact specimen is the steepest, and its strength is the highest. That’s to say, the hidden fissure weakens the strength and stiffness of specimens.

The peak stress of *α* = 90° specimen is 15.64 MPa, close to 16.04 MPa of the intact specimen, and the peak stress of other specimens is much lower. The maximum peak strain occurs to *α* = 30° specimen, followed by the *α* = 0° specimen. The peak strain of other hidden-fissured specimens is similar. To summarize, the horizontal fissure has the lowest effect on specimen strength and stiffness.

#### Failure mode

**Figure 5:**
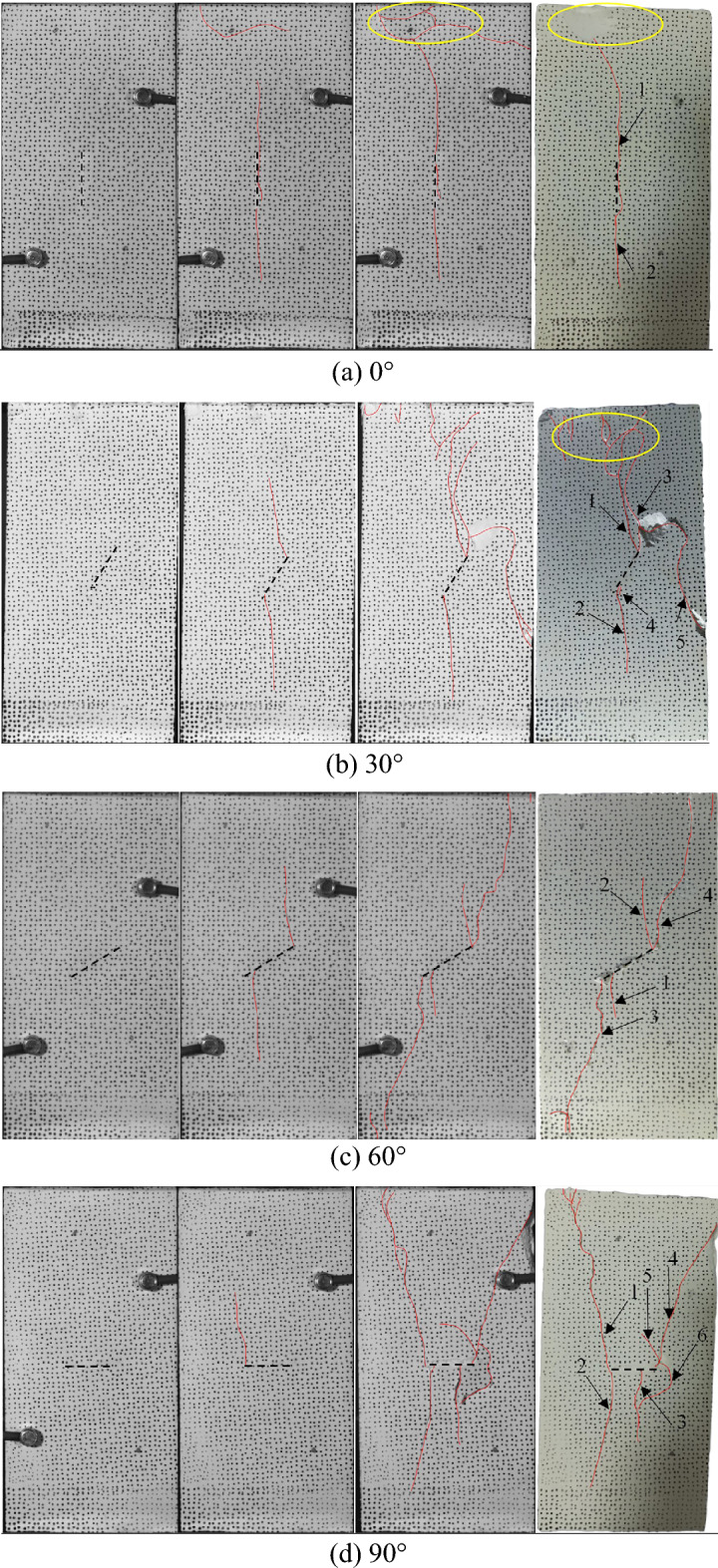
Failure morphology of hidden-fissured specimens changes with fissure inclination angles.

Figure 5 shows the failure morphology of hidden-fissured specimens with different inclination angles under uniaxial compression stress. The black dash line represents the hidden fissure and the red line represents the crack.

For *α* = 0° hidden-fissured specimen (Fig. 5a), the crack is first generated at the upper and lower tips of the prefabricated hidden fissure and extends to both ends of the specimen. After crack 1 is initiated, it grows and turns direction to the left during of propagating upward, and at the same time, it extends down through the hidden fissure, accelerating the opening of the hidden fissure. Finally, crack 1 continues to expand to connect with crack 2 until failure. Multi-crack coalescence occurs in far-field areas, and local surface peeling is observed.

For *α* = 30° hidden-fissured specimen (Fig. 5b), two wing cracks (crack 1 and crack 2) initiate firstly at the two tips of the prefabricated hidden fissure. With the increase of stress, two secondary coplanar cracks, crack 3 and crack 4, are generated at the fissure tip. They keep growing along the hidden fissure and then turn to the direction of the maximum principal stress. Due to the development of crack 3 and the increase of stress, crack 5 appears, and the specimen shear-damaged.

For *α* = 60° hidden-fissured specimen (Fig. 5c), wing cracks 1 and 2 are first initiated, and then cracks 3 and 4 are generated. Crack 1 does not start from the fissure tip. Crack 2 starts from the upper tip of the hidden fissure, and the crack propagation path is relatively straight after initiation. After cracks 1 and 2 are generated, there is a tendency to extend to the middle of specimen ends. Both cracks 3 and 4 are generated at the fissure tips and extend to the corner of the specimen. The propagation paths of these two cracks are tortuous, and fine particles are observed on crack surfaces. Bulges and blocks are observed around the hidden fissures. The hidden fissures are tensile-damaged when the stress is small, and then shear dislocation occurs as the stress increases. The hidden fissures, crack 3 and crack 4 form a weak surface, resulting in the final failure.

For *α* = 90° hidden-fissured specimen (Fig. 5d), crack 1 is firstly generated at the left tip of the hidden fissure and extends upward. Then, crack 2 starts at the left tip and extends downward. Crack 3 is generated in the middle right area of the prefabricated hidden fissure. Then crack 4 is generated in the tip region on the right side of the prefabricated hidden fissure and extends upward, and cracks 5 and 6 are initiated and propagate to the middle zone. Meanwhile, the surface near the hidden fissure is peeled off. The main reason may be that the hidden fissure is compressed under perpendicular stress, making the surrounding zone of the hidden fissure more broken.

#### Deformation characteristics

**Figure 6:**
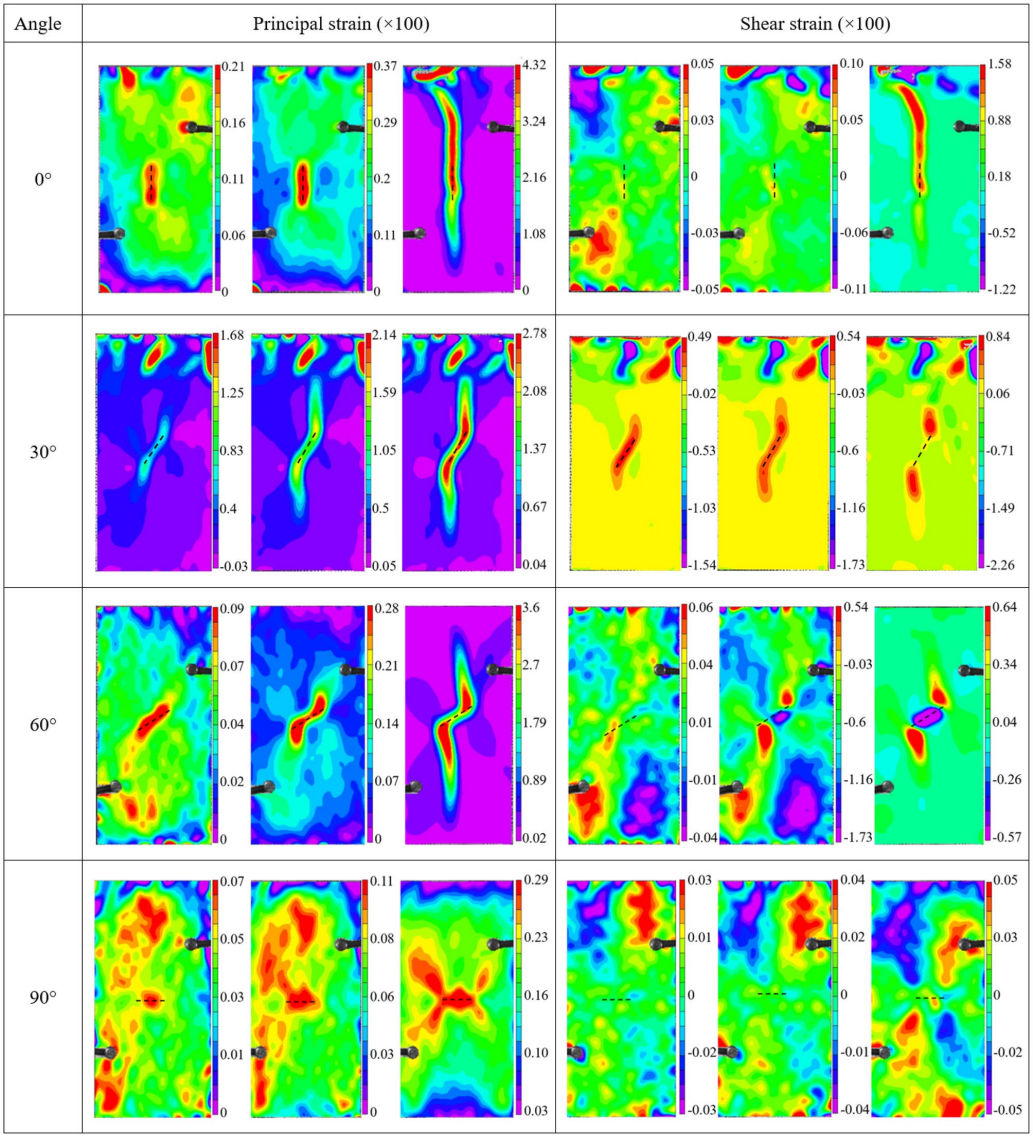
Strain nephogram of hidden-fissured specimens with different inclination angles.

As a non-contact monitoring technology, DIC has become a widely used technique in experimental mechanics. The basic principle of DIC is to record the digital speckle patterns during the deformation process by a high-speed camera. Two speckle patterns are calculated to obtain the surface strain field, and the initiation and propagation of cracks can be further recognized and characterized. The principal strain and shear strain nephograms of hidden-fissured specimens with different inclination angles are calculated, as shown in Fig. 6.

For *α* = 0° hidden-fissured specimen, the principal strain nephogram matches well with the actual crack path. The principal tensile strain concentrates around the fissure and gradually extends as the stress increases. Meanwhile, a shear strain concentration band is formed when the stress is large enough. What’s more, the value of tension strain is larger than shear stress in the third stage. For *α* = 0° hidden-fissured specimen, tensile damage opening the fissure dominates its deformation process, and shear damage also contributes significantly to final failure.

For *α* = 30° hidden-fissured specimen, principal strain values are generally greater than shear strain. The shear stress on the *α* = 30° fissure is larger than the tensile stress, the bond between particles breaks under shear stress to form cracks, and crack surfaces move further away under tensile stress.

Similarly, tensile strain concentrates on the hidden fissure for *α* = 60° hidden-fissured specimen. However, the distribution regularities of shear stress are different. The wing cracks are subject to the tensile-shear combined effect, and the center area of the hidden fissure is subject to the compression shear effect.

The situation of *α* = 90° hidden-fissured specimen is most remarkable. Tensile strain concentrates on the hidden fissure and gradually spreads to the crack path, while the shear strain distributes along the 45° direction. This is similar to the stress evolution characteristics of intact specimens and consistent with the above findings that the horizontal hidden fissure has the minimum effect on specimen failure.

### Close-fissured specimens

#### Stress–strain relationship

**Figure 7:**
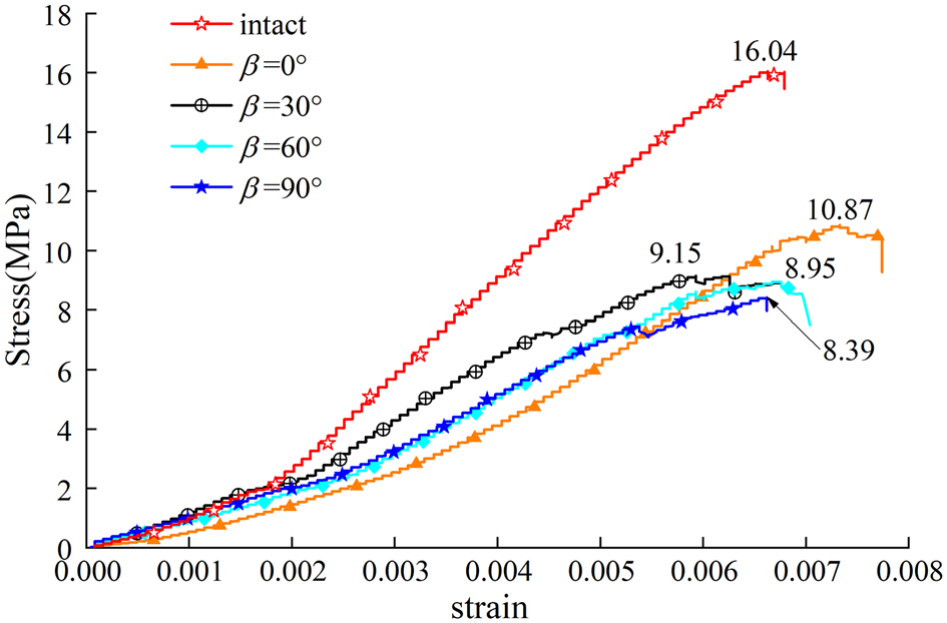
Stress–strain curves of close-fissured specimens.

Figure 7 shows the stress–strain curves of the intact specimens and close-fissured specimens with different inclination angles. The slope of the linear elastic stage of the intact specimen is larger than that of other close-fissured specimens. The strength of the close-fissured specimens is much lower than that of the intact specimen. The stress–strain curves of *β* = 0°, 30° and 60° close-fissured specimens fluctuate around the peak points, while the curve of *β* = 90° close-fissured specimen directly dropped. The peak stress and strain values of *β* = 0° close-fissured specimen are the highest, while the maximum strain values of the other three specimens are close. The curves of the *β* = 60° and *β* = 90° specimens almost overlap in the linear elastic stage, and the two curves deviate due to stress suddenly drop induced by cracks behaviors.

#### Failure mode

**Figure 8:**
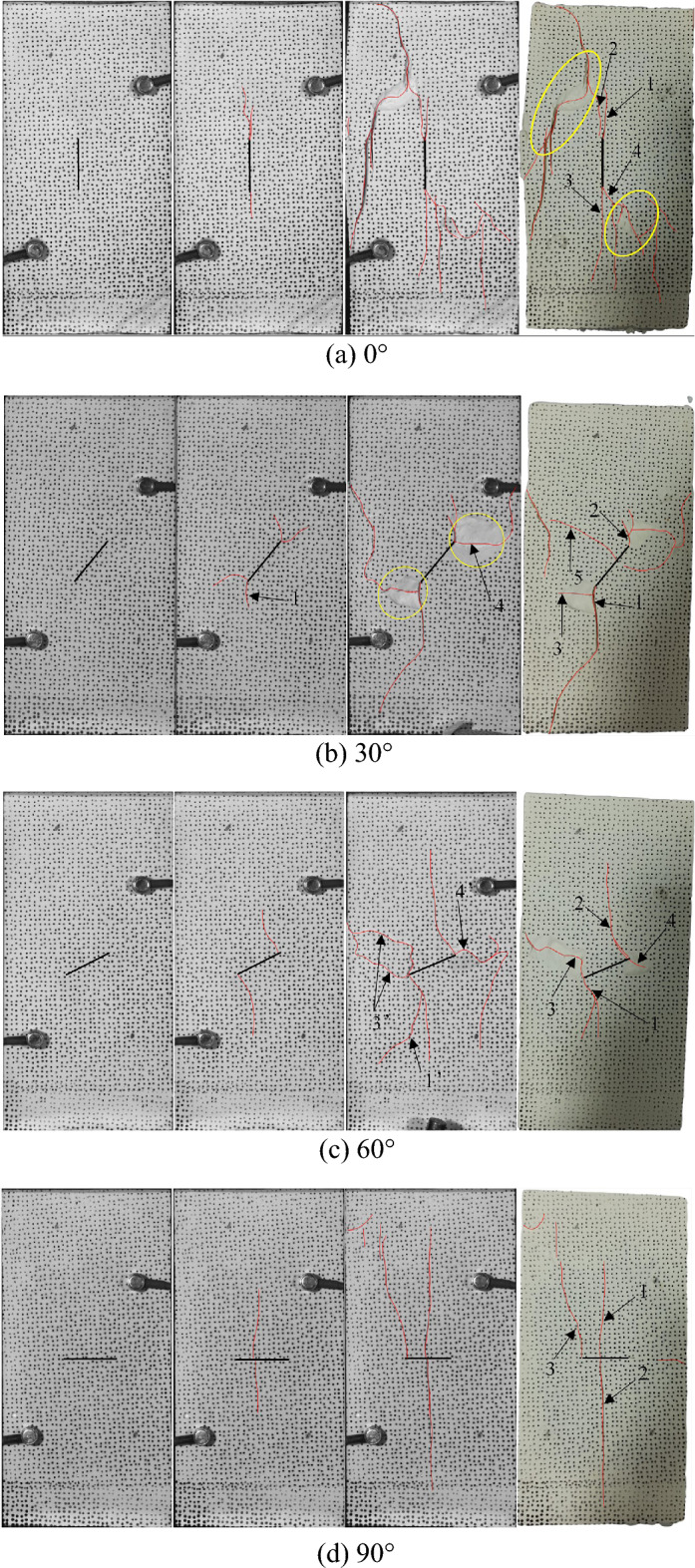
Failure morphology of close-fissured specimens with different inclination angles.

Figure 8 shows the failure morphology of close-fissured specimens with different inclination angles under uniaxial compression stress, indicating the failure process and the final failure morphology of the specimens. For example, when *β* = 60°, wing cracks (cracks 1 and 2) are first generated at the fissure tip and extend to the maximum principal stress direction. The anti-wing cracks (cracks 3 and 4) were also initiated at fissure tips later. Finally, the specimen loses the bearing capacity due to the closure of the closed fissure, which is different from the failure of the *α* = 60° hidden-fissured specimen. By comparison, it is found that the wing crack propagation length of the hidden-fissured specimen is shorter, and cracks are generated inside the prefabricated fissure rather than the tip. Anti-wing cracks are also observed in the close-fissured specimens, which are not found during the cracking of hidden-fissured specimens. The hidden-fissured specimen is sliding failure due to the weak plane formed by cracks penetration, and the close-fissured specimen is losing the bearing capacity due to the large displacement induced by the closure of the closed fissures. For example, crack development is relatively simple for *β* = 90° close-fissured specimen. Cracks 1 and 2 first initiate in the middle of the prefabricated closed fissure and extend to both ends of the specimen, then crack 3 is generated at the left fissure tip and extends to the end of the specimen. The width of crack 1 and crack 2 increases with the increase of load. Finally, the middle part of the prefabricated closed fissure is contact. The reason may be that the central crack makes the two sides of the crack surface form a cantilever-like state, and the compression bending is formed. Obviously, the failure mode of *β* = 90° close-fissured specimen is quite different from the failure mode of *α* = 90° hidden-fissured specimen.

By comparing and analyzing the fracture modes of the hidden- and close-fissured specimens, the crack shape is depicted in Table 1. For *α* = 0° hidden-fissured specimen, the initial crack starts from fissure tips, the crack length of the hidden-fissured specimen is longer than that of the close-fissured specimen. When the inclination angle is 30°, secondary cracks appear at closed fissure tips, which do not appear in the hidden-fissured specimen. Meanwhile, the cracks propagate longer than those in the close-fissured specimen. When the inclination angle is 60°, the cracks in the hidden-fissured specimen develop longer, while the arc of the wing crack is not as flexural as that of the close-fissured specimen. When the inclination angle is 90°, the crack initiates from the hidden fissure tip, while the crack of the close-fissured specimen usually initiates at the middle position of the fissure, indicating significant differences in failure mechanism. In general, the crack propagation length of the hidden-fissured specimen is longer than that of the close-fissured specimen. The reason may be that the crack of the close-fissured specimen has not yet fully developed, and closure of the closed fissure leads to large displacement and loss of bearing capacity.

**Table 1:** Cracks comparison of hidden-fissured and close-fissured specimens.

Angle (°)	0	30	60	90
Hidden fissure				
Closed fissure				

#### Deformation characteristics

**Figure 9:**
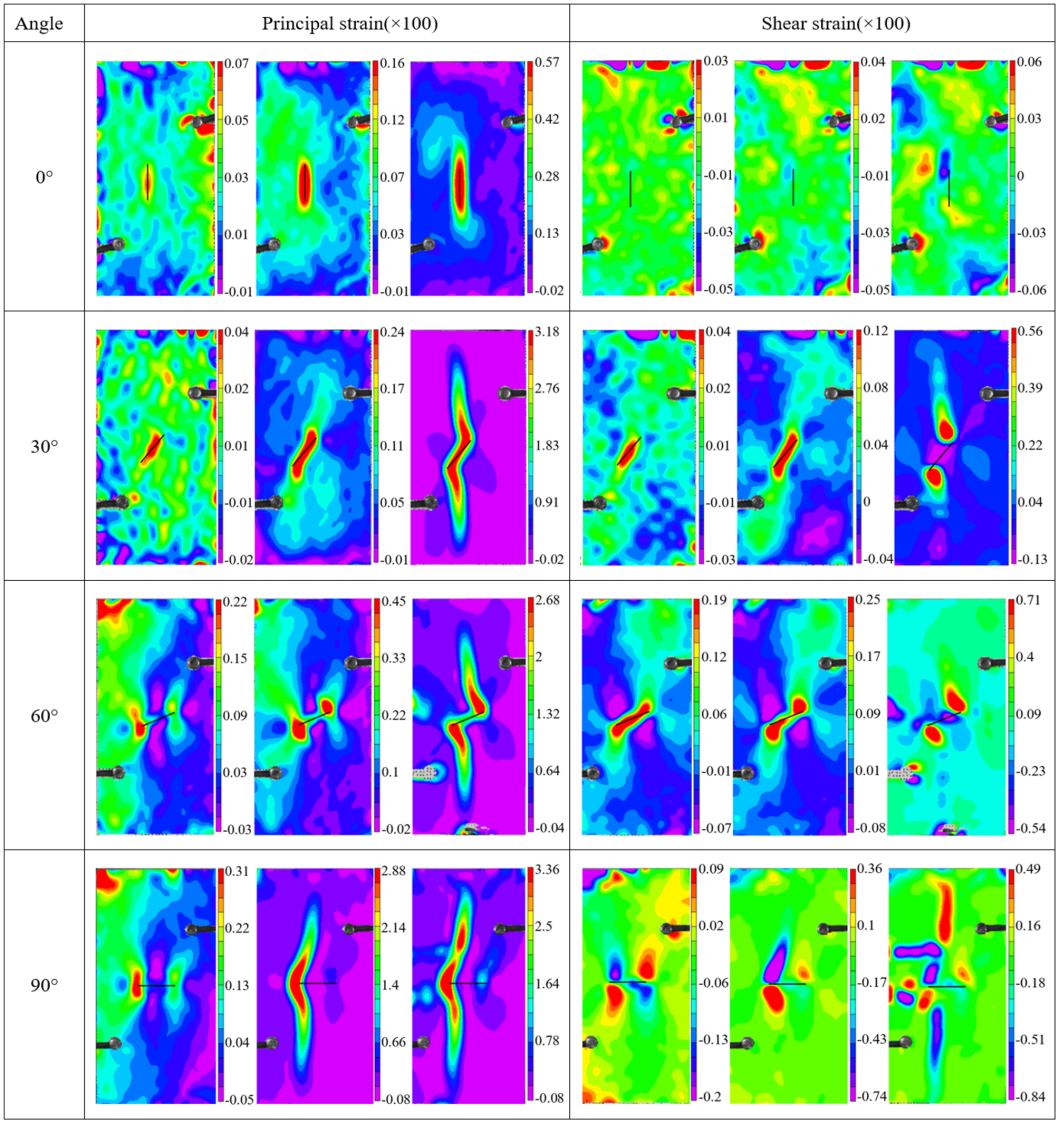
Strain cloud diagram of close-fissured specimens with different inclination angles.

The principal strain and shear strain nephograms of close-fissured specimens with different inclination angles are shown in Fig. 9. The solid black line in the figure indicates the location of the closed fissure.

For *β* = 0° close-fissured specimen, the principal strain concentrates around the closed fissure while the shear strain disperses in the whole specimen. Deformation of *β* = 0° close-fissured specimen is mainly controlled by tensile stress.

For *β* = 30° close-fissured specimen, both tensile and shear deformation occur to the specimen in the elastic stage. The tensile-shear effect is observed at closed fissure tips and controls the final failure, while compression-shear appears at the closed fissure. Compared with the *α* = 30° hidden fissure specimen, the tensile strain values are more considerable. Closed fissures can lead to large deformation and strain.

For *β* = 60° close-fissured specimen, it is noted that the principal strain concentration band only appears along wing cracks instead of the closed fissure, which is different from *α* = 60° hidden-fissured specimen. Shear sliding along the fissure surface induces shear strain.

For *β* = 90° close-fissured specimen, it is found that the strain at the left tip of the fissure is greater than that at the right tip. Two wing cracks initiation possess asynchronism, the left side starts first, and the right side follows. What’s more, different from *α* = 90° hidden-fissured specimen, shear strain band appears along the cracking path.

## Discussion

### Mechanical properties

**Figure 10:**
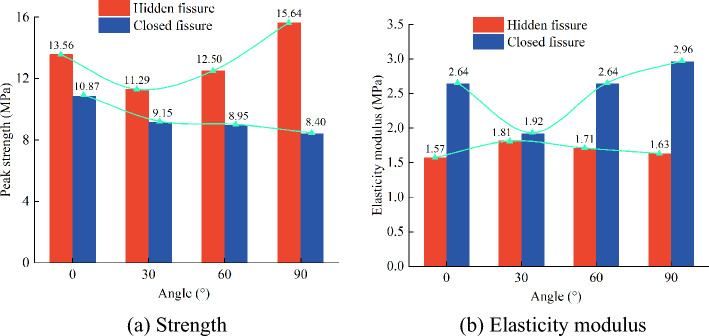
Comparison of the mechanical parameters of the hidden-fissured and close-fissured specimens.

As shown in Fig. 10, the evolutions of the unconfined compressive strength values of the hidden-fissured and close-fissured specimens changing with inclination angles are compared. There are similarities, as well as differences, between hidden- and close-fissured specimens.

First, it should be noted that the strength of all fractured specimens is lower than the intact specimen (16.04 MPa), indicating that the hidden fissures can weaken material strength, while the weakening effect is smaller than the closed fissures regardless of the fissure angle. Second, when the hidden fissure is horizontal, its effect on reducing strength and stiffness is the lowest. The strength and elasticity modulus of hidden-fissured specimens show a U-shaped trend, and the lowest values occur to *α* = 30° situation; the strength of the close-fissured specimens decreases with the angle increases, while the horizontal closed fissure weakens most.

### Cracking mechanism

**Figure 11:**
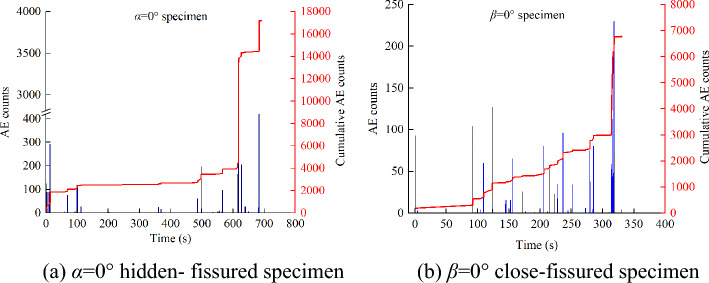
AE counts-time-cumulative AE counts of specimens.

AE technology was used to monitor the cracking behaviors in real-time. The AE counts-time-cumulative AE counts of the hidden-fissured *α* = 0° and close-fissured *β* = 0° specimens are taken as examples (Fig. 11). As shown in Fig. 10, the cumulative AE counts possess similar evolution trends, i.e., slowly increase at first, steadily grow as stress increases, and sharp soar before failure. The evolution trend of AE counts is helpful for forecast specimen oncome broken, while the RA–AF relationship is adopted to analyze the failure mechanism of different specimens.

Studies have proved that the relationship between RA and AF is quite different when tensile fracture or shear fracture occurs^26–28^. The RA–AF parameters are defined as:

1 $$RA = \frac{Risetime}{{Amplitude}}$$

2 $$AF = \frac{Counts}{{Duration}}$$

**Figure 12:**
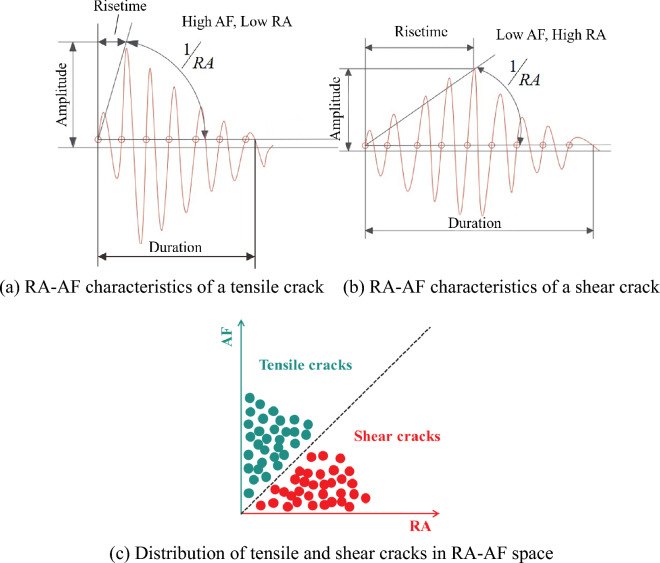
Typical crack classification based on RA and AF.

From the waveform perspective, tensile cracks form an AE waveform with high energy and high amplitude, but a short Risetime, thus the tensile cracks appear in the upper left region (Fig. 12a,c). The peak value of the waveform generated by shear failure has a longer delay from the first arrival point of the P wave, and the AE signal has a longer Risetime with a longer Duration, but a lower average frequency, thus the shear cracks appear in the lower right region (Fig. 12b,c). Consequently, the RA–AF relationship based on acoustic emission characteristics can effectively reveal rock cracking mechanism^29^.

**Figure 13:**
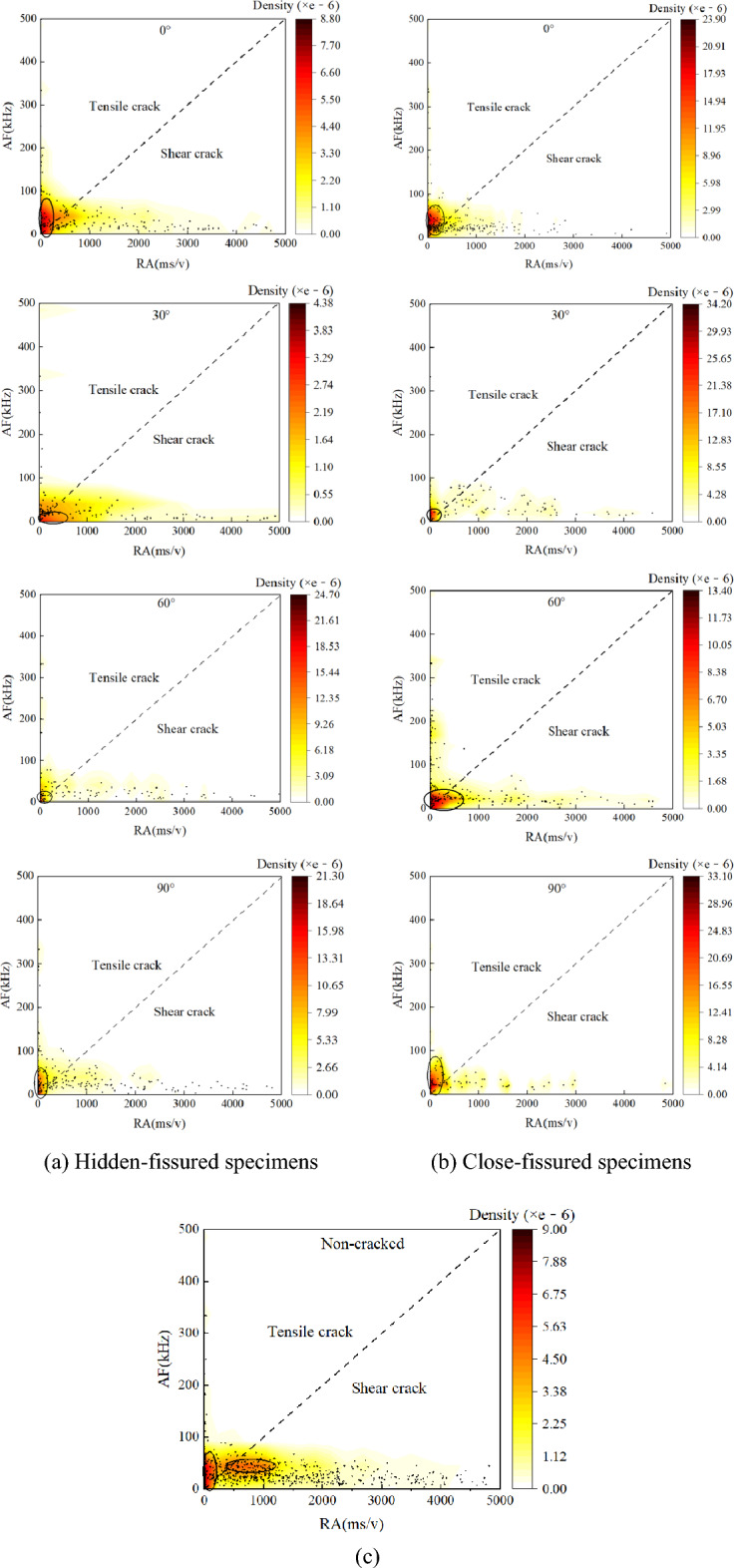
The RA–AF distribution of specimens.

Figure 13 shows the RA–AF distribution of intact, hidden- and close-fissured specimens, indicating significant differences in failure mechanism. The color represents the scatter density of RA–AF points, as the color from white to red (and even black) means that the density gradually increases.

For the intact specimen (Fig. 13c), both tensile and shear cracks are dense, and the quantity ratio is 42.50%:58.49%. Shear damage occurs easier than tensile cracks, which is consistent with the failure process. Figure 13a is the RA–AF diagram of the hidden-fissured specimen. When *α* = 0°, the quantity ratio of tensile and shear cracks is 55.92%:44.08%. The extremely high AF and RA values are relatively small, and most of the scatter points are concentrated near the coordinate origin and close to the AF axis. As the coordinate value increases, the number of scatter points gradually decreases. The core area of the scattered point distribution, that is, most of the black circle area, is above the dotted line and close to the AF axis, indicating that the fracture mode of the hidden-fissured specimen is mainly tensile fracture. When *α* = 30°, the quantity of shear cracks (60.92%) is higher than tensile cracks (39.08%). Meanwhile, the scatter distribution is quite different from the *α* = 0° specimen. Most of the RA–AF scatters are close to the RA axis, and the points along the RA axis become more and more sparse with the increase of the coordinate value. Most of the core area is located below the dotted line and close to the RA axis, indicating that the failure of the specimen is dominated by shear cracks, supplemented by tensile cracks. When *α* = 60°, the quantity ratio of tensile and shear cracks is 56.49%:43.51%. It can still be found that most of the points are concentrated near the origin of the coordinates. In general, there are more points above the dotted line, indicating that the failure of the specimen is mainly tensile failure. When *α* = 90°, the quantity ratio of tensile and shear cracks is 61.30%:38.70%. The scatter distribution is similar to those of the *α* = 0° specimen, but the total number of scatters is less, and the core area is mainly located above the black dotted line, indicating that the fracture mode of the specimen is still dominated by tensile fracture mode, supplemented by shear failure.

Figure 13b is the RA–AF diagram of the close-fissured specimen. When *β* = 0°, the quantity ratio of tensile and shear cracks is 61.15%:38.85%. The points are observed to extend along the RA axis. The area with the largest scatter density is above the dotted line and close to the AF axis. The RA value is much larger than ten times AF, indicating that the tensile crack is stronger than the shear crack in the fracture mode of the close-fissured specimen. When *β* = 30° and *β* = 90°, the quantity ratio of tensile and shear cracks is 63.37%:36.63% and 62.22%:37.78%. Most of the points are concentrated near the origin of the coordinate axis, and the core area is above the black dotted line and close to the AF axis. The specimen is a tensile-shear mixed fracture mode dominated by tensile fracture. When *β* = 60°, the quantity ratio of tensile and shear cracks is 58.33%:42.67%. The scatter is nearly half-distributed on both sides of the black dotted line, and the core area is half above the dotted line and half below the dotted line, indicating that it is a tensile-shear mixed fracture mode.

In general, the number of RA–AF scatter points generated by the hidden-fissured specimen is more than that of the close-fissured specimen, and most scatter points are concentrated near the origin of the coordinate axis. The area with the largest scatter density is mostly close to the AF axis, except for the *α* = 30° case. The fracture mode of the close-fissured specimens is a tensile-shear mixed fracture mode, and that of the hidden-fissured specimens is mainly a tensile-shear mixed fracture mode dominated by tensile fracture.

## Conclusions

The cracking behaviors and failure mechanism of the hidden-fissured specimens are studied by comparing them with intact and close-fissured specimens. Through uniaxial compression tests, combined with rock AE real-time monitoring and DIC optical photograph, the influences of hidden fissures on the mechanical parameters, cracking process, fracture mode and failure mechanism are studied. The study supports the following conclusions:

1. Hidden fissures significantly weakened the strength compared with the intact specimen, while the deterioration effect is weaker than the close-fissured specimens. The differences are induced by structural properties, i.e., the material particles inside the hidden fissure provide internal cohesion, instead of only friction existing on the fissure surface of close-fissured specimens. Among all fissure angles, the *α* = 90° hidden fissure has the lowest influence on specimen strength and stiffness.
2. Similar to the closed-fissured specimens, wing cracks and secondary cracks were observed for the hidden-fissured specimens. However, the initiation position of specimens containing horizontal fissures was different due to stress field distribution, indicating that the influences of hidden fissures on the cracking morphology of specimens are different from that of closed fissures.
3. The RA–AF relationship based on acoustic characteristics is investigated to reveal the failure mechanism. The failure of the close-fissured specimens is mainly the tensile-shear mixed fracture mode, while failure of the hidden-fissured specimens is mainly the tensile fracture mode and supplemented by the shear.

## Data Availability

The datasets used and/or analysed during the current study available from the corresponding author on reasonable request.
